# Fast recovery of disrupted tip links induced by mechanical displacement of hair bundles

**DOI:** 10.1073/pnas.2016858117

**Published:** 2020-11-16

**Authors:** R. G. Alonso, M. Tobin, P. Martin, A. J. Hudspeth

**Affiliations:** ^a^HHMI, The Rockefeller University, New York, NY 10065;; ^b^Laboratory of Sensory Neuroscience, The Rockefeller University, New York, NY 10065;; ^c^Laboratoire Physico-Chimie Curie, Institut Curie, PSL Research University, CNRS UMR168, F-75248 Paris, France;; ^d^Sorbonne Université, F-75252 Paris, France

**Keywords:** auditory system, cadherin, cochlea, hair cell, vestibular system

## Abstract

Hearing and balance rely on the capacity of mechanically sensitive hair bundles to transduce vibrations into electrical signals that are forwarded to the brain. Hair bundles possess tip links that interconnect the mechanosensitive stereocilia and convey force to the transduction channels. A dimer of dimers, each of these links comprises two molecules of protocadherin 15 (PCDH15) joined to two of cadherin 23 (CDH23). The “handshake” that conjoins the four molecules can be disrupted in vivo by intense stimulation and in vitro by exposure to Ca^2+^ chelators. Using hair bundles from the rat’s cochlea and the bullfrog’s sacculus, we observed that extensive recovery of mechanoelectrical transduction, hair bundle stiffness, and spontaneous bundle oscillation can occur within seconds after Ca^2+^ chelation, especially if hair bundles are deflected toward their short edges. Investigating the phenomenon in a two-compartment ionic environment that mimics natural conditions, we combined iontophoretic application of a Ca^2+^ chelator to selectively disrupt the tip links of individual frog hair bundles with displacement clamping to control hair bundle motion and measure forces. Our observations suggest that, after the normal Ca^2+^ concentration has been restored, mechanical stimulation facilitates the reconstitution of functional tip links.

## Significance

Each of the sensory receptors responsible for hearing or balance—a hair cell—has a mechanosensitive hair bundle. Mechanical stimuli pull upon molecular filaments—the tip links—that open ionic channels in the hair bundle. Loud sounds can damage hearing by breaking the tip links; recovery by replacement of the constituent proteins then requires several hours. We disrupted the tip links in vitro by removing the calcium ions that stabilize them, and then monitored the electrical response or stiffness of hair bundles to determine whether the links could recover. We found that tip links recovered within seconds if their ends were brought back into contact. This form of repair might occur in normal ears to restore sensitivity after damage.

Our ability to hear sounds and detect accelerations stems from the capacity of specialized cells to transduce mechanical energy into electrical signals that are interpreted by the nervous system. Transduction is accomplished by mechanoreceptors known as hair cells that—with subtle morphological and physiological variations—operate in fundamentally the same way among all vertebrates (1–3).

Each hair cell detects mechanical stimuli with an organelle, the hair bundle, composed of tens to hundreds of rod-like, actin-filled stereocilia that protrude from the cuticular plate, an actin-rich structure at the cell’s apical surface (4). These stereocilia are arranged in a staircase, increasing in length monotonically toward a single true cilium termed the kinocilium (5). In the mammalian cochlea, the kinocilium degenerates after the hair bundle has developed, but the organelle persists in hair cells from the mammalian vestibular system and those from other vertebrates. Upon deflection of the hair bundle, the stereocilia pivot about their insertions into the cuticular plate; the resultant shear between contiguous stereocilia modulates the extension of elastic elements that are coupled to mechanoelectrical-transduction channels, eliciting an electrical response (6, 7). By controlling the resting tension of these gating springs, adaptation motors set the channels’ open probability and thus regulate the sensitivity of mechanoelectrical transduction (8–10).

Each stereocilium bears an oblique filament—the tip link—that connects its tip to the flank of a neighbor in the taller stereociliary row and is thought to be a component of the gating spring (11–14). A tip link consists of a parallel homodimer of cadherin 23 (CDH23) in its upper two-thirds and a parallel homodimer of protocadherin 15 (PCDH15) in its lower third (15). Each of the 38 unique extracellular cadherin domains in the two proteins is stabilized in part by binding of Ca^2+^ to sites at one or both of the domain’s ends (16).

Ca^2+^ also stabilizes the molecular handshake that interconnects the proteins at their amino termini (17). Exposure of a hair cell to a Ca^2+^ chelator for as little as a few seconds disrupts these interactions and terminates mechanoelectrical transduction (18, 19). Although the handshake interaction might potentially be regenerated after the restoration of Ca^2+^, there are at least three reasons why such recovery might not occur. First, each tip link in a resting hair bundle bears a resting tension on the order of 10 pN (8, 20). When a link is severed, the two ends are expected to undergo elastic retraction from one another. The second issue is that the tension in intact tip links pulls a hair bundle toward its short edge by exerting force against the flexion of the stereociliary pivots. When all of the tip links of a frog’s hair bundle are disrupted, the bundle can lunge more than 100 nm in the positive direction (18). The stiffer hair bundles of the rat’s cochlea move somewhat less (8). As a result of the geometric relationship between the motion of a hair bundle and the shear between contiguous stereocilia, the larger movement would displace the separated ends of a tip link by up to 20 nm. A final possibility is that dissociated cadherins are internalized and therefore no longer available to reconstitute tip links (21). In the present investigation, we inquired whether tip links might recover if their separated ends were reapposed, for example by deflecting a hair bundle well in the negative direction before internalization could occur.

## Results

### Rapid Recovery of Mechanoelectrical Transduction upon Hair Bundle Deflection.

**Fig. 1.**
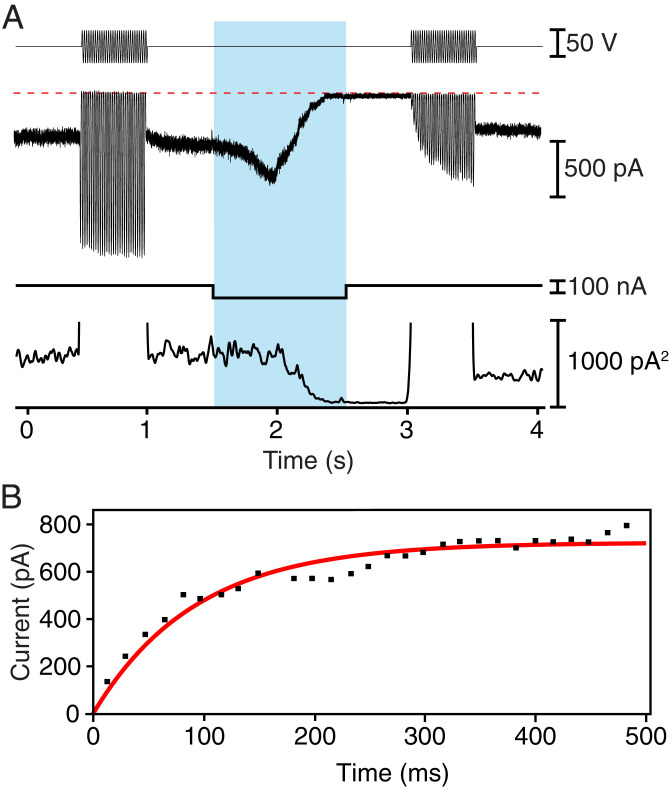
Rapid recovery of mechanoelectrical transduction in outer hair cells from the rat’s cochlea. (*A*) A fluid-jet stimulator was driven at 60 Hz with two sinusoidal stimulus trains (top trace), one before and one after an iontophoretic current pulse (bottom trace) that released EDTA. The pale blue band here and in subsequent figures delineates the period of iontophoresis. The transduction current (second trace) was initially large but fell to nearly zero after iontophoresis before recovering more than one-half of its original magnitude during the second stimulus train. The variance of the transduction current (third trace) fell during iontophoresis as transduction was interrupted, but recovered partially after a second epoch of stimulation. The abscissa represents zero variance. (*B*) For the record shown in *A*, the recovery of the transduction current after the iontophoretic pulse followed an exponential relation (red line) with a time constant of 100 ms.

To determine whether tip link integrity can be restored on a short timescale after disruption, we first measured the mechanoelectrical transduction currents from outer hair cells of an excised preparation of the neonatal rat’s cochlea before and after disrupting the tip links. We used a calibrated fluid jet to deflect each hair bundle, voltage clamping to measure the transduction current, and iontophoresis to deliver EDTA onto the hair bundle. We found that in response to a 60-Hz sinusoidal stimulus that deflected the hair bundle by ∼100 nm, the transduction current could recover in part within 1 s after the iontophoretic pulse. In one example (Fig. 1*A*), comparison of the responses measured before tip link disruption and after recovery indicated that the transduction current achieved 57% of its original level. For this cell, the mean and variance of the transduction current reached 72% and 49% of their respective control values. The time course of the recovery was roughly exponential with a time constant of 100 ms (Fig. 1*B*). Nine of the 16 outer hair cells that we examined displayed recovery of their transduction currents during sinusoidal stimulation to at least 100 pA, or 20 to 60% of the original level.

These observations could not be explained by incomplete disruption of the tip links: Before the second stimulus train, both the mean and the variance of the resting current were negligible, reflecting the absence of mechanotransduction. Furthermore, the time evolution of the current during the iontophoretic pulse reflected the known response of the transduction machinery to a Ca^2+^ chelator (8, 19): The current first increased owing to a rise in tip link tension, and then fell to zero as the links were disrupted and the transduction channels closed. The variability in recovery likely reflected differences in the number of tip links that were reconstituted before the component cadherins moved away or were subducted from the membrane.

### Rapid Recovery of Mechanical Properties upon Hair Bundle Deflection.

Electrical recording in a low-Ca^2+^ environment that emulates endolymph is difficult. Moreover, we ascertained that iontophoresis of a Ca^2+^ chelator does not disrupt tip links in solutions with Ca^2+^ concentrations in the millimolar range. The mechanical properties of the hair bundle offered an alternative manifestation of the disruption and recovery of tip links. We therefore designed a series of protocols to examine how the process occurs. The fragility of the mammalian cochlea largely precludes an ex vivo preparation that recreates the ionic milieu in which the cochlea normally operates. The two-compartment preparation of the bullfrog’s sacculus, however, reconstitutes the ionic environment of hair cells and retains most of the active characteristics of the cells (22, 23). In addition, the high cohesiveness of bullfrog hair bundles ensures that they move as a unit in response to mechanical stimulation, which facilitates the interpretation of stiffness measurements (6). We therefore elected to investigate the recovery process further with that preparation.

We attached the tip of a flexible glass fiber to the kinociliary bulb and moved the fiber’s base sinusoidally at 10 Hz with an amplitude of 100 nm. We then evaluated the displacement of the hair bundle as an estimate of tip link integrity. Tip links contribute considerably to the total stiffness of a hair bundle (8, 18–20). For hair bundles of the bullfrog’s sacculus, as an example, tip link disruption by Ca^2+^ chelators decreases the stiffness from ∼1,200 to 200 μN·m^−1^ (19). The residual stiffness is attributed to the actin filaments at the stereociliary pivots, the basal insertions of the stereocilia (24).

**Fig. 2.**
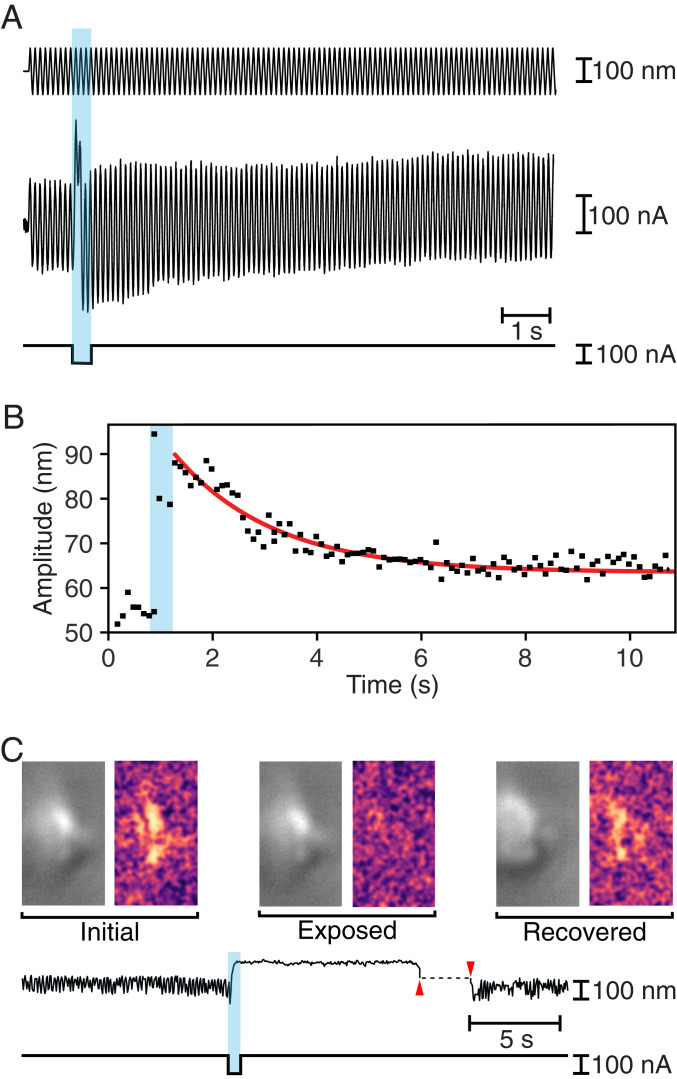
Rapid recovery of mechanical properties in hair bundles of the bullfrog’s sacculus. (*A*) While a 10-Hz sinusoidal stimulus of amplitude 100 nm (top trace) was delivered to the base of a flexible glass fiber, a pulse of iontophoretic current (bottom trace) released EDTA. The hair bundle’s movement (middle trace) increased immediately after iontophoresis, but returned toward the initial value over a few seconds. (*B*) For the record shown in *A*, the decline in the amplitude of hair bundle oscillation after the iontophoretic pulse followed an exponential relation (red line) with a time constant of 1,910 ms. (*C*) To the left in each pair of images in the top row are individual frames of a movie of a spontaneously oscillating hair bundle (Movie S1) representing the unperturbed bundle (Initial), the same bundle after exposure to EDTA (Exposed), and finally the bundle after transient displacement in the negative direction (Recovered). To the right are three images, each obtained by subtracting the original frame from the subsequent frame. The Initial and Recovered images reveal spontaneous hair bundle motion, which is absent in the Exposed image. The time course of the hair bundle’s position in the movie (upper trace) shows suppression of the spontaneous oscillations during iontophoretic application of EDTA (lower trace) and their recovery after the bundle was pushed in the negative direction (between the red arrowheads).

The mechanical responses of bullfrog hair bundles were consistent with the observations from the rat’s outer hair cells. Before an iontophoretic pulse, stimulation typically resulted in sinusoidal oscillations 50 nm in amplitude. Immediately after the iontophoretic pulse, the amplitude of oscillation increased to ∼90 nm, then progressively declined toward the initial value before reaching a plateau near 60 nm (Fig. 2*A*). The decrease in the amplitude of the oscillations followed an exponential trajectory with a time constant of about 1,900 ms (Fig. 2*B*). The change in the amplitude of the oscillations implied that the stiffness of the hair bundle fell to 55% of its control level immediately after iontophoresis. By the end of the recording, however, the bundle regained 83% of its original stiffness.

When exposed to physiologically appropriate ionic solutions, hair bundles of the frog’s sacculus oscillate spontaneously (22). These movements emerge from the interplay between negative stiffness and the adaptation machinery (25). Because spontaneous oscillations require the normal operation of the transduction apparatus, disrupting the tip links would be expected to arrest the movements, which might resume if the tip links were healed.

To examine this possibility, we selected hair bundles that displayed large spontaneous oscillations that were readily recognized upon microscopic observation. Upon iontophoresis of EDTA, each hair bundle underwent an abrupt positive displacement and the oscillations stopped. Left to itself in a control experiment, the bundle remained quiescent for minutes. If instead we used a stiff glass fiber to displace the hair bundle in the negative direction for a few seconds, the oscillations promptly resumed (Fig. 2*C* and Movie S1). This result is consistent with the reconstitution of tip links and the recovery of mechanoelectrical transduction.

### Stiffness Recovery as an Indication of Tip Link Reattachment.

We next used displacement-clamp feedback to control the bundle’s position and to monitor the force necessary to maintain that position. With a flexible fiber attached to the kinociliary bulb of an individual hair bundle, we iontophoretically delivered EDTA to selectively disrupt the tip links. This approach allowed us to apply various displacement protocols and to assess the stiffness of a hair bundle throughout an experiment.

**Fig. 3.**
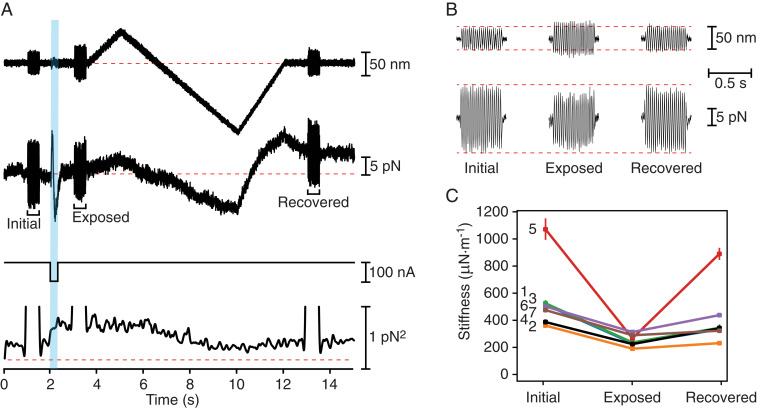
Facilitation of hair bundle recovery in the bullfrog’s sacculus by mechanical displacement. (*A*) In a displacement-clamp experiment, a feedback system imposed a ramp displacement on a hair bundle (first trace), moving the bundle first in the positive direction to prevent prompt recovery, and then more extensively in the negative direction. At three times during this paradigm, a 500-ms epoch of ±25-nm, 40-Hz sinusoidal stimulation was superimposed on the displacement-command signal. An iontophoretic pulse (third trace) released EDTA to break tip links. The force (second trace) necessary to clamp the bundle at the outset (Initial) diminished after exposure to iontophoretically applied EDTA (Exposure) but recovered almost completely by the experiment’s end (Recovered). The variance of the force (fourth trace) confirmed the bundle’s softening after iontophoresis and its recovery during the negative phase of the ramp. The dashed line represents the background noise. (*B*) Enlarged records of the hair bundle displacement (top traces) and clamp force (bottom traces) from *A* demonstrate that maintaining an oscillation of similar—or even greater—magnitude required less force shortly after iontophoresis. (*C*) Data from seven hair cells, which are numbered as in Table 1, reveal a significant decrease (*P* < 0.01 by a single-sided paired *t* test) in hair bundle stiffness after iontophoretic pulses. The stiffness then recovered significantly (*P* < 0.05 by the same test) following negative hair bundle displacements. The bundle whose responses are depicted in *A* and *B* is number 4; SDs are shown when they exceed the size of the data points.

As expected, the displacement clamp kept the amplitude of hair bundle movement relatively constant during sinusoidal stimulation, but the force required to do so decreased after exposure of the bundle to Ca^2+^ chelator (Fig. 3 *A* and *B*). Because clamping was incomplete, the bundle’s excursion also increased somewhat. Both changes implied a greater compliance of the bundle owing to the disruption of tip links. For seven hair bundles from six preparations, the stiffness after chelation fell to 230 ± 16 μN·m^−1^ (mean ± SEM; Table 1), a value comparable to the stiffness of the stereociliary pivots (19). This result implies that tip link disruption was nearly complete.

**Table 1.** Stiffness recovery by bullfrog hair cells

Hair cell no.	Initial stiffness, μN·m^−1^	Stiffness after EDTA exposure, μN·m^−1^	Stiffness after recovery, μN·m^−1^	Endolymph Ca^2+^ concentration, μM
1*	490 ± 26	211 ± 13	311 ± 14	25
2*	339 ± 17	171 ± 14	211 ± 10	25
3*	501 ± 24	217 ± 12	322 ± 15	250
4	365 ± 19	205 ± 11	320 ± 26	250
5	1,039 ± 85	246 ± 12	860 ± 53	250
6	478 ± 17	292 ± 10	415 ± 16	250
7	452 ± 13	269 ± 11	302 ± 14	250

Each stiffness was estimated by measuring the flexion of a flexible glass fiber attached at the hair bundle’s tip during sinusoidal stimulation. Asterisks indicate bundles subjected to step displacements; the remainder were displaced with ramps. Values are reported as means ± SDs for 21 determinations. For the entire sample, the stiffness decreased significantly after exposure with respect to the initial value (*P* < 0.007). After the negative displacements, the stiffness recovered significantly with respect to the exposed value (*P* < 0.04). If we disregard the cell (number 5) with an exceptionally high stiffness, the corresponding values show still greater significance (*P* = 0.0001 and *P* < 0.002, respectively).

To foster the possible reformation of tip links, we then displaced each hair bundle as much as −150 nm with a slow displacement ramp. Moving the bundle toward its short edge—a negative stimulus—would be expected to bring the tips of successive ranks of stereocilia closer together and might therefore promote the reassociation of tip link cadherins. Indeed, upon stimulation after the displacement, the force required during sinusoidal stimulation attained nearly its control level (Fig. 3 *A* and *B*). A similar result was obtained with a step displacement (*SI Appendix*, Figs. S1 and S2). The hair bundle stiffness therefore recovered substantially (Table 1). Comparison of the stiffnesses before and after treatment showed an average recovery of 73.5 ± 4.4% (mean ± SEM; *n* = 7). The recovery of 81.0 ± 4.9% (mean ± SEM; *n* = 4) during a ramp exceeded that of 63.4 ± 0.6% (mean ± SEM; *n* = 3) for a step.

The variance in the force applied to the hair bundle provided an additional, qualitative indication of the change in a bundle’s stiffness during and after the disruption of tip links and indicated when tip link recovery occurred (Fig. 3*A*). For the same seven hair bundles, the variance in hair bundle force increased from a control value of 0.47 ± 0.01 pN^2^ to 0.92 ± 0.10 pN^2^ (mean ± SEM) immediately after an EDTA pulse. During the ramp protocol, the variance remained high until the hair bundle was pushed in the negative direction, whereupon it fell progressively to a plateau of 0.53 ± 0.01 pN^2^ (mean ± SEM), a value slightly exceeding that prior to the iontophoretic pulse.

As a result of separation between the ends of the cadherin molecules in a disrupted tip link, recovery should be less likely if a hair bundle remains in its resting position or is moved in the positive direction. Consistent with that expectation, recovery was never observed in a hair bundle displaced in the positive direction, nor was any change noted during control experiments in which no chelator was applied (*SI Appendix*, Fig. S3). These observations confirmed that the changes in stiffness resulted from the joint action of disrupting the tip links with a Ca^2+^ chelator and displacing the stereocilia in the negative direction.

### Negative Hair Bundle Movement during Exposure to Ca^2+^ Chelators.

The sequence of hair bundle forces associated with the disruption and regeneration of tip links revealed unexpected complexity in recordings from bullfrog hair cells. In six of the seven cells examined, there was a sustained positive offset of 20.1 ± 7.0 pN (mean ± SEM) at the end of the stimulation protocol with respect to the value before EDTA exposure (*SI Appendix*, Note S1). In the absence of displacement clamping, bundles displayed a corresponding offset in displacement that occurred even after the kinocilium had been dissected free of the clustered stereocilia (*SI Appendix*, Note S2 and Figs. S4 and S5). The basis of this phenomenon is uncertain.

### Repeated Recovery of Stiffness by a Hair Bundle.

**Fig. 4.**
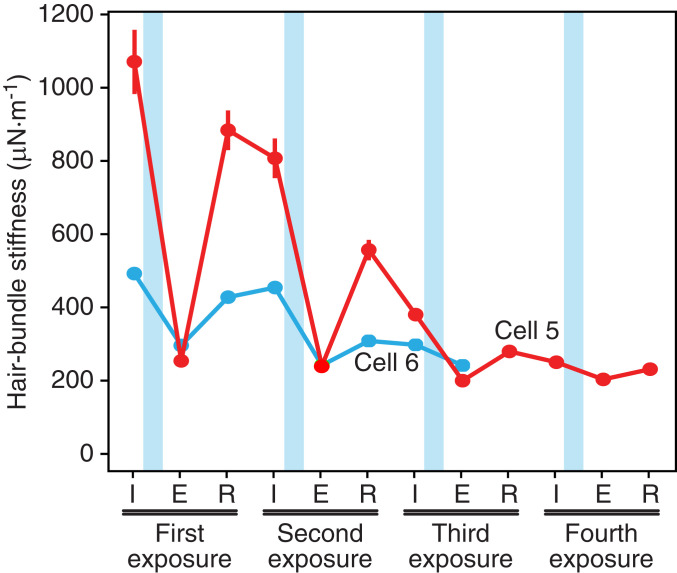
Sequential disruption and recovery of tip links in the bullfrog’s sacculus. For two hair bundles also described in Table 1, successive applications of EDTA reduced the stiffness to approximately that associated with the stereociliary pivots. When subjected to ramp displacements, one bundle recovered part of its stiffness at least three times and the other twice. The data points represent the initial stiffness (I), that just after EDTA exposure (E), and that following recovery (R).

In two experiments, we examined the possibility that tip links can recover after successive treatments with Ca^2+^ chelator. By applying to the same hair bundle sequential ramp protocols separated by 10 to 20 s, we were able to obtain some degree of tip link recovery after as many as three cycles of iontophoresis (Fig. 4). In each instance, the recovery was partial, so the stiffness declined progressively toward the value associated with the stereociliary pivots. After the bundle’s stiffness had reached that level, no further recovery occurred. Repeated recovery of the transduction current was also observed in one hair cell of the rat’s cochlea through five cycles of iontophoresis.

### Resting Tension in Tip Links.

The resting tension of tip links was first estimated for the bullfrog’s saccular hair cells maintained in a homogeneous ionic environment (20). The present experiments afforded an opportunity to make corresponding measurements on unrestrained, oscillating hair bundles in a two-compartment chamber with more physiologically appropriate saline solutions bathing the apical and basal cellular surfaces.

In a resting hair bundle, the force exerted by tip link tension is equal and opposite that owing to the flexion of the stereociliary pivots. By measuring the movement *X*_SP_ of each bundle upon disruption of its tip links, one may therefore calculate the average tension *t*_TL_ along the oblique axis of each tip link as follows:

$$t_{\mathrm{TL}}=\frac{K_{\mathrm{SP}}X_{\mathrm{SP}}}{\gamma N_{\mathrm{TL}}},$$ [1]

in which *K*_SP_ = 250 μN·m^−1^ represents the stiffness of the stereociliary pivots; *γ* = 0.14, the geometrical gain factor; and *N*_TL_ = 40, the number of tip links. Because a healthy hair bundle in a two-compartment chamber usually oscillates spontaneously, we measured *X*_SP_ as the bundle’s displacement from the midpoint between the positive and negative extremes of its oscillation (*SI Appendix*, Fig. S6). For 13 oscillatory hair bundles, we found *X*_SP_ = 462 ± 93 nm (mean ± SEM) and estimated the average tension in the tip links of an unstimulated hair bundle as *t*_TL_ = 20.6 ± 4.1 pN (mean ± SEM). Even in a two-compartment environment, some hair bundles did not oscillate, possibly as a result of mechanical damage or Ca^2+^ loading during the dissection in standard saline solution. Quiescent hair bundles showed smaller movements upon EDTA iontophoresis, *X*_SP_ = 187 ± 24 nm (mean ± SEM; *n* = 31). If the number of tip links remained constant, this implies a lower tip link tension, *t*_TL_ = 8.3 ± 1.1 pN. Tip links in oscillatory hair bundles thus appeared to bear more than twice the tension borne by those in quiescent bundles.

## Discussion

Tip link disruption is a well-characterized form of hair cell damage (26). Intense hair bundle stimulation caused by prolonged exposure to loud sounds can damage tip links in vivo (27), whereas Ca^2+^ chelators can dissociate them in vitro (18, 19). This damage can be partially reverted in 12 h to 48 h as the tip links regenerate and restore mechanosensitivity through a two-step mechanism (21, 28–31). Interacting PCDH15 molecules first form temporary tip links that partially restore transduction but not adaptation. The upper portions of the tip links are then replaced with CDH23 to restore normal transduction (21).

The complete replacement of tip links is both metabolically costly and relatively slow. In this study, we have shown that tip links can recover within seconds after disruption by Ca^2+^ chelation. This phenomenon constitutes an unusual form of repair for a molecular lesion: disrupted links evidently reconstitute themselves from their components. One possible utility of the recovery process is the restoration of hair bundle function after exposure to injurious events such as loud sounds (26, 27). Tip links might accordingly act as security releases that prevent more extensive damage to a hair bundle owing to overstimulation (32).

The rapidity of the recovery suggests that the phenomenon involves the reconstitution of the original tip links rather than the mobilization of stored cadherin molecules or the synthesis of new ones. There are two plausible mechanisms of recovery that are not mutually exclusive. It seems most likely that chelation disrupts the molecular handshake between PCDH15 and CDH23 dimers, and that after the restoration of Ca^2+^ the amino termini simply diffuse until they collide and reconstitute the handshake. By approximating the free ends of the PCDH15 and CDH23 dimers, pushing a hair bundle in the negative direction facilitates this process. An alternative possibility is that the handshake is never disrupted, but that the softening of tip links during Ca^2+^ chelation reflects the unfolding of extracellular cadherin domains. Such an event would profoundly affect a tip link: Each 4-nm domain includes a total of about 35 nm of polypeptide chain, so the unfolding of only a few domains would result in a greatly elongated structure with a far lower stiffness. According to this model, the recovery upon the restoration of Ca^2+^ would reflect the refolding of the cadherin domains, which for PCDH15 monomers occurs within seconds in the absence of tip link tension (13).

Under conditions in which the hair bundles oscillated spontaneously, our measured value for the positive movement of bullfrog hair bundles following EDTA iontophoresis was as much as threefold that obtained earlier (18). The difference likely reflects the fact that the present data were obtained in a two-compartment recording chamber, so that the stereocilia were bathed in low-Ca^2+^ endolymph and the cell somata in perilymph, rather than both surfaces in a homogenous medium. Calcium ions allow the adaptation motors of hair cells to slip down the stereocilia (33) and might have reduced tip link tension in the previous study. As a consequence of the greater movements in the present experiments on oscillatory hair bundles, our estimate of the tension in individual tip links is more than twice that reported for recordings for quiescent hair bundles in a homogeneous solution (20) and resembles that for outer hair cells at the apex of the rat’s cochlea (8). Moreover, the reported value for tip link tension is a minimal estimate: We assumed a total of 40 tip links in each hair bundle, nearly the maximum possible number for a large bundle, when in reality some bundles were smaller and some links were likely broken during dissection.

## Methods

Detailed methods are provided in *SI Appendix, Materials and Methods*.

### 

#### Electrical recording from mammalian hair cells.

The sensory epithelia of young rats and mature bullfrogs were maintained under microscopic observation in species-appropriate physiological saline solutions. While mechanoelectrical transduction of each outer hair cell from the rat’s cochlea was monitored by whole-cell, tight-seal recording, the hair bundle was displaced sinusoidally by fluid-jet stimulation. After tip links had been disrupted by the iontophoretic application of EDTA, the electrical response was recorded for indications of recovery.

#### Mechanical recording from anuran hair cells.

Each hair bundle from the frog was displaced with an elastic glass fiber driven by a piezoelectric stimulator. In some experiments, sinusoidal force stimulation allowed assessment of the bundle’s stiffness during and after the iontophoretic application of EDTA. In other instances, a hair bundle was displacement-clamped and the force was recorded. In both instances, the restoration of a bundle’s mechanical properties provided an index of tip link recovery.

## Supplementary Material

Supplementary File

Supplementary File

## Data Availability

All study data are included in the article and *SI Appendix*.
