# Supplementary material for: Fast recovery of disrupted tip links induced by mechanical displacement of hair bundles
Source: Proc Natl Acad Sci U S A. 2020 Nov 16;117(48):30722–7. doi: 10.1073/pnas.2016858117 (PMC7720144; doi:10.1073/pnas.2016858117)
Supplement: Supplementary File [file pnas.2016858117.sapp.pdf]

Supporting Information for

Fast recovery of disrupted tip links induced by mechanical displacement of hair bundles

R. G. Alonso [a], M. Tobin [b,c,*], P. Martin [b,c] and A. J. Hudspeth [a,1]

[a] Howard Hughes Medical Institute and Laboratory of Sensory Neuroscience, The Rockefeller University, New York, NY 10065; [b] Laboratoire Physico-Chimie Curie, Institut Curie, PSL University, CNRS UMR168, Paris, France; [c] Sorbonne Université, Paris, France

Corresponding author: hudspaj@rockefeller.edu

This file contains:

- Materials and Methods
- Notes S1 and S2
- Figs. S1 to S6
- Video S1
- References

www.pnas.org/cgi/doi/10.1073/pnas.2016858117

SI Materials and Methods

Preparation of the rat's cochlea

The procedures were conducted at the Institut Curie and were approved by the Ethics Committee in accordance with the European and French National Regulation for the Protection of Vertebrate Animals used for Experimental and other Scientific Purposes (Directive 2010/63; French Decree 2013-118).

Experiments were performed on excised cochleae of Sprague-Dawley rats (*Rattus norvegicus,* Janvier Labs) of both sexes and 7-10 days of age. The dissection and isolation of the cochleae followed a published procedure (1,2). After a rat had been euthanized and decapitated, the inner ears were extracted from the head. Each cochlear bone was carefully opened and the membranous cochlear duct uncoiled from the modiolus. After excision of the cochlear partition, the stria vascularis was removed and the tectorial membrane gently peeled away. An apical or middle turn of the organ of Corti was positioned under nylon fibers in an experimental chamber containing artificial perilymph (150 mM Na^+, 6 mM K^+, 1.5 mM Ca^{2+}, 159 mM Cl^-, 10 mM Hepes, 8 mM D-glucose, and 2 mM sodium pyruvate; pH 7.4; 315 mOsmol·kg^{-1}). During the experiment, we used perfusion to change the hair bundles' ionic environment to a variant (150 mM Na^+, 6 mM K^+, 3.3 mM Ca^{2+}, 163 mM Cl^-, 4 mM HEDTA, 10 mM Hepes, 8 mM D-glucose, and 2 mM sodium pyruvate) with a free Ca^{2+} concentration of 22 μM.

Preparation of the bullfrog's sacculus

The procedures were conducted at The Rockefeller University and at the Institut Curie with the approval of the respective Institutional Animal Care and Use Committees.

Experiments were performed on hair cells from adult bullfrogs (*Rana catesbeiana*) of both sexes. After an animal had been euthanized, the sacculi were carefully removed by a standard protocol (3). Each saccular macula was sealed with tissue adhesive (Vetbond, 3M) across a 1 mm hole centered on a 10 mm square of aluminum foil. The foil

was situated in a two-compartment chamber with the macular side of the sacculus facing upward. The lower compartment was filled with oxygenated artificial perilymph (114 mM Na^+, 2 mM K^+, 2 mM Ca^{2+}, 118 mM Cl^-, 5 mM Hepes, and 3 mM D-glucose; pH 7.4; 230 mOsmol·kg^{-1}). The apical surface of the hair cells was exposed for 35 min at room temperature to 67 mg·L^{-1} of protease (type XXIV; Sigma) to loosen the otolithic membrane, which was carefully removed with an eyelash. The upper compartment was then filled with oxygenated artificial endolymph (2 mM Na^+, 118 mM K^+, 250 µM Ca^{2+}, 118 mM Cl^-, 5 mM Hepes, and 3 mM D-glucose; pH 7.4; 230 mOsmol·kg^{-1}).

Measurement of hair-bundle position

Experiments on both preparations were conducted with similar apparatus. Each preparation was placed on an upright microscope (BX51WI, Olympus) and the hair cells were visualized with a 60X, water-immersed objective lens of numerical aperture 0.9 and differential-interference-contrast optics. Rat hair cells were observed during experiments with a charge-coupled-device camera (LCL-902K, Watec). Video observations of the bullfrog's sacculus videos were conducted after an additional 4X magnification with a CMOS camera (DCC3240M, Thorlabs) or a high-speed video camera (ZYLA-5.5-CL10-W, Andor).

To record a hair bundle's position, the preparation was illuminated with a 630 nm light-emitting diode (UHP-T-SR, Prizmatix) and the resultant shadow was projected onto a dual photodiode at a magnification of 1300X. The output of the photodiode was low-pass filtered at 2 kHz with an eight-pole anti-aliasing filter (Benchmaster 8.13, Kemo). The photodiode was calibrated by translating the bundle's image through a succession of 10 µm steps with a mirror mounted on a piezoelectric actuator (PA 120/14 SG, Piezosystem Jena). Digital data samples were acquired at intervals of 200 µs.

Mechanical stimulation with fluid jets

Because they are complexly shaped and poorly cohesive, hair bundles from outer hair cells of the rat's cochlea are difficult to stimulate with glass fibers. We therefore deflected each bundle with a fluid jet driven by a piezoelectric disk, which recruited all the stereocilia (1). When viewed under the objective lens of the microscope in the plane of the sensory epithelium, the tip of each pipette was positioned along the axis of mirror symmetry of each hair bundle at a 8 μm distance on the bundle's abneural side. Liquid exiting the pipette therefore displaced the stereocilia towards their shortest row.

Mechanical stimulation with flexible fibers

Owing to the strong attachments among the stereocilia of a hair bundle from the bullfrog's sacculus, force applied to the kinocilium uniformly displaces all the stereocilia (4). We accordingly used a flexible glass fiber attached to the kinociliary bulb to mechanically stimulate the hair bundle.

Each flexible fiber was displaced by a piezoelectric actuator (PA 4/12, Piezosystem Jena) positioned with a micromanipulator (MP-285, Sutter Instruments) and driven by an amplifier (ENV 800, Piezosystem Jena). The fiber was forged from a borosilicate capillary (1B120F-3, World Precision Instruments). After the capillary had been tapered with an electrode puller (P-2000, Sutter Instruments), its tip was melted with a platinum filament and pulled laterally with a 120 V solenoid to form a 90° angle to the shaft. The resultant fiber was approximately 100 μm in length and 1 μm in diameter. The fiber was sputter-coated with gold-palladium (Hummer 6.2, Anatech) to increase its optical contrast. To enhance the coupling of the stimulus fiber to the kinociliary bulb, we submerged the fiber's tip in a droplet of 200 $mg \cdot L^{-1}$ concanavalin A for 15 min before an experiment.

Each fiber's stiffness and drag coefficient were estimated by measuring the Brownian motion of its tip in water. We then obtained parameter values by fitting the power

spectrum of the displacement to a Lorentzian relation (5). The fibers in this study had stiffnesses of 160-380 $\mu N \cdot m^{-1}$ and drag coefficients of 150-290 $nN \cdot s \cdot m^{-1}$; they behaved as first-order, low-pass filters with cut-off frequencies near 200 Hz.

Displacement clamping

We used negative feedback to control the position of a hair bundle according to a computer-generated external command (1,6,7). By doing so, we were able to monitor the force required to hold a hair bundle stationary or to deflect it to a desired position. The computer's sampling interval of 200 μs set an upper limit on the potential frequency response of the system, but a eight-pole, low-pass Bessel filter (Benchmaster 8.07, Kemo) imposed a cutoff at 2 kHz between the computer's output and the stimulator's input to ensure stability.

Use of the displacement-clamp system and sinusoidal stimulation allowed us to measure the decrease and subsequent recovery of hair-bundle stiffness with good temporal resolution. However, this approach confronted an inevitable problem: because the response time of the clamp system is finite, responses of progressively higher frequency become progressively less well clamped. The clamp's settling time constant was generally about 2 ms, which corresponded to a corner frequency near 80 Hz. By selecting a stimulus frequency of 40-50 Hz, we accepted some non-ideality in clamping in the interest of improved frequency resolution in stiffness measurements.

The force F_{SF} exerted by the stimulus fiber against a hair bundle was estimated from the positions of the fiber measured at its base and at its tip (8):

$$F_{SF} \simeq 0.97 \mathrm{K}_{SF}(Y - X) - 0.94 \lambda_{SF} \dot{X} - 0.57 \lambda_{SF} \dot{Y} \ , \qquad (1)$$

in which K_{SF} and λ_{SF} represent respectively the stiffness and hydrodynamic friction coefficient of the stimulus fiber, Y the displacement of its base, and X the displacement of its tip. $\dot{Y}$ and $\dot{X}$ are the time derivatives of the corresponding variables. Because the stimulus frequencies were well below the cut-off frequency of the fiber, this low-frequency

approximation of the periodic force applied by the fiber is expected to be accurate (8). Positive movements and forces were those directed toward a hair bundle's tall edge.

The stiffness K_{HB} of each hair bundle was estimated by measuring the average force F_{SF} and displacement X_{HB} for 21 successive periods of sinusoidal stimulation. The stiffness was then computed for each sinusoidal train as

$$K_{HB} = \mathrm{F}_{SF}/\mathrm{X}_{HB} \ . \quad (2)$$

Voltage-clamp recording

We recorded mechanoelectrical-transduction currents of outer hair cells of the rat cochlea with whole-cell, tight-seal electrodes. Each micropipette was pulled (P-97, Sutter Instruments) from a thick-walled capillary (1B150F-4, WPI) and fire-polished to obtain a tip 2-3 μm in diameter. The electrode was filled with intracellular solution (142 mM Cs^{+}, 11 mM Na^{+}, 3.5 mM Mg^{2+}, 149 mM Cl^{-},1 mM EGTA, 5 mM ATP, 0.5 mM GTP, and 10 mM Hepes; pH 7.3; 295 $mOsmol·kg^{-1}$) and contained a chlorinated silver electrode. When immersed in standard saline, the micropipette had a resistance of 1.5-4 MΩ. The voltage across each hair cell's membrane was controlled and currents were recorded with an amplifier (Axopatch 200B, Axon Instruments). The cell was held at a potential of -80 mV. The voltage offset was corrected before forming a gigaohm seal with a cell and the pipette's capacitance was compensated to achieve a cut-off frequency of 1-9 kHz. Current signals were low-pass filtered at 1.25-12.5 kHz and sampled at intervals of 40-400 μs.

Iontophoresis

We used iontophoretic pulses to deliver Ca^{2+} chelators in the vicinity of the hair bundles. Microelectrodes were fabricated from borosilicate glass capillaries (TW 120-F, World Precision Instruments) with an electrode puller (P-80/PC, Sutter Instruments) and filled with 500 mM EDTA in 1 M NaOH. We used a current amplifier (Axoclamp 2B, Axon Instruments) to control the release of EDTA. A holding current of 10 nA kept EDTA from

diffusing into the endolymphatic solution, and pulses of -100 nA released the chelator. The electrodes' tips were directed at and situated about 2 μm from tops of the hair bundles.

SI Notes

Note S1. Negative hair-bundle movement during exposure to Ca^{2+} chelators

The sequence of hair-bundle forces associated with the breaking and regeneration of tip links reveals unexpected complexity in recordings from bullfrog hair cells. In six of the seven cells, there was a sustained positive offset of 20.1 ± 7.0 pN (mean ± SEM) at the end of the stimulation protocol with respect to the value before EDTA exposure (Fig. 3*A*; *SI Appendix,* Fig. S4). This force offset was absent when tip links were not broken.

In principle, this tensioning of the hair bundle would be compatible with increased activity of the adaptation machinery (7,9). A decrease in the cytoplasmic Ca^{2+} concentration after tip-link rupture would cause the adaptation motors to ascend in the stereocilia and thus generate a negative offset in the position of the hair bundle after tip-link recovery. Nevertheless, this effect was probably masked by the presence of another, more intriguing phenomenon: a negative movement of the hair bundle that occurred seconds after tip-link breakage.

Upon exposure to Ca^{2+} chelator there was a sudden increase in the force that reflected a rise in tip-link tension, followed by the abrupt decrease that resulted from tip-link rupture. Although these observations accorded with previous studies (8,10,14,15), the traces also revealed a subsequent rebound in the force (Fig. 3*A*). The force exerted by the fiber indicated that the displacement clamp acted to counter a negative movement of the hair bundle (*SI Appendix,* Fig. S4).

Although never observed in outer hair cells from the rat's cochlea, this unexpected effect was present to a certain degree in most recordings from the two-compartment preparation of the bullfrog's sacculus. Because this preparation recreated the environment in which hair cells normally operate, it differed from the homogeneous ionic environment of previous investigations of tip-link breakage (1,6,10,11). To determine whether the unreported negative movement was consistently associated with tip-link

rupture in this preparation, we measured the position of the top stereociliary row and kinocilium in unrestrained, oscillating hair bundles and applied iontophoretic pulses of Ca^{2+} chelator of various durations. Although some hair bundles responded to Ca^{2+} sequestration with canonical dynamics—a rapid negative twitch followed by a large positive displacement to a stable level—others showed a negative rebound in position (*SI Appendix,* Fig. S4). Nine of 18 spontaneously oscillating bundles displayed some degree of negative movement, ranging from -5 nm to -485 nm and averaging -166 ± 70 nm (mean ± SEM). For an additional 15 of 31 quiescent bundles that displayed negative movements, the magnitude averaged -183 ± 36 nm (mean ± SEM). This effect was most prominent when the duration of the iontophoretic pulse exceeded a few seconds, during which the negative displacement reached a plateau that variously lay either positive or negative to the bundle's initial position. After reaching a stable plateau, the hair bundle never returned to its initial position. Moreover, because the bundle displayed a reduced stiffness and never responded to another epoch of chelator iontophoresis, the phenomenon did not result from recovery and tensioning of the tip links.

The negative hair-bundle movement often observed after tip-link disruption by Ca^{2+} chelation—or the corresponding positive force measured under displacement-clamp conditions—remains to be explained. One possibility is that the cuticular plate deforms in such a way as to alter the forces within the stereociliary cluster. For hair cells of the bullfrog's sacculus, the cuticular plate is concave upward, a configuration that pushes the stereociliary tips together (12,13). If the curvature of the cuticular plate were to increase after tip-link breakage, the stereocilia of the longest rank would be expected to undergo a negative displacement.

Note S2. Lack of contribution of the kinocilium to negative movements

Aside from those associated with tip links, what other forces might act on a hair bundle? Each bundle possesses a single kinocilium that bears an axoneme with dynein motors

(14). Because the kinocilium can be motile (15,16), it might exert a force that affects the hair bundle's position. To test that possibility, we separated the kinocilium from an oscillating hair bundle and usually held its tip several micrometers away from the stereociliary cluster with a glass microelectrode (17). Because the optical contrast of a bundle with a detached kinocilium was too low to allow the use of a photodiode, we used video microscopy to record the position of the hair bundle and a tracking algorithm (18) to trace independently the positions of the tallest stereociliary row and of the kinocilium (*SI Appendix*, Fig. S5*A*). We were then able to measure both displacements before, during, and after breaking the tip links with EDTA. Even with the kinocilium separated from and moving independently of the stereociliary cluster, four of the five hair bundles tested displayed a negative movement following EDTA exposure (*SI Appendix,* Fig. 5*B*). In some instances, the negative motion proceeded in rapid steps of irregular size, a phenomenon that occurred even when the dissociated kinocilium was immobilized against the epithelial surface by a microelectrode (*SI Appendix,* Fig. 5C). The negative hair-bundle movements thus stem from a source other than the kinocilium.

SI Figures and Legends

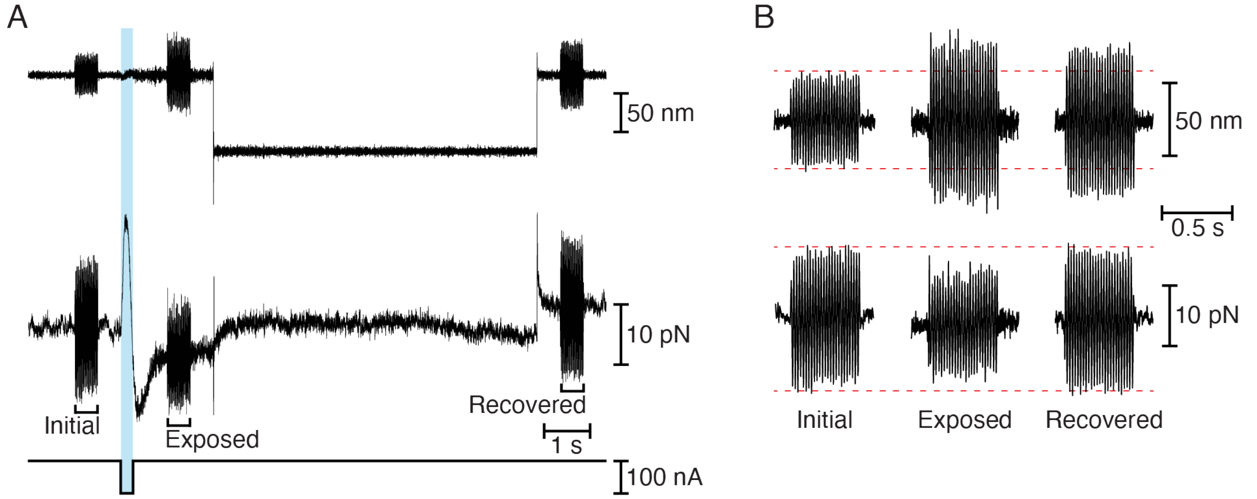

Fig. S1. Step protocol for facilitating tip-link recovery in bullfrog saccular hair cells. (*A*) The displacement-clamp protocol imposed a step displacement of the bundle (first trace) following the iontophoretic pulse (third trace). The force necessary to clamp the bundle (second trace) diminished after iontophoresis but recovered almost completely by the experiment's end. At three times a 500 ms epoch of ±25 nm, 50 Hz sinusoidal stimulation was superimposed on the displacement-command signal. To display the meaningful parts of the data at an appropriate scale, the transient upstrokes and downstrokes at the onset and offset of the force step have been reduced. (*B*) Enlarged records of hair-bundle displacements (top traces) and clamp forces (bottom traces) during sinusoidal stimulation highlight the phenomenon of diminished and recovered hair-bundle stiffness.

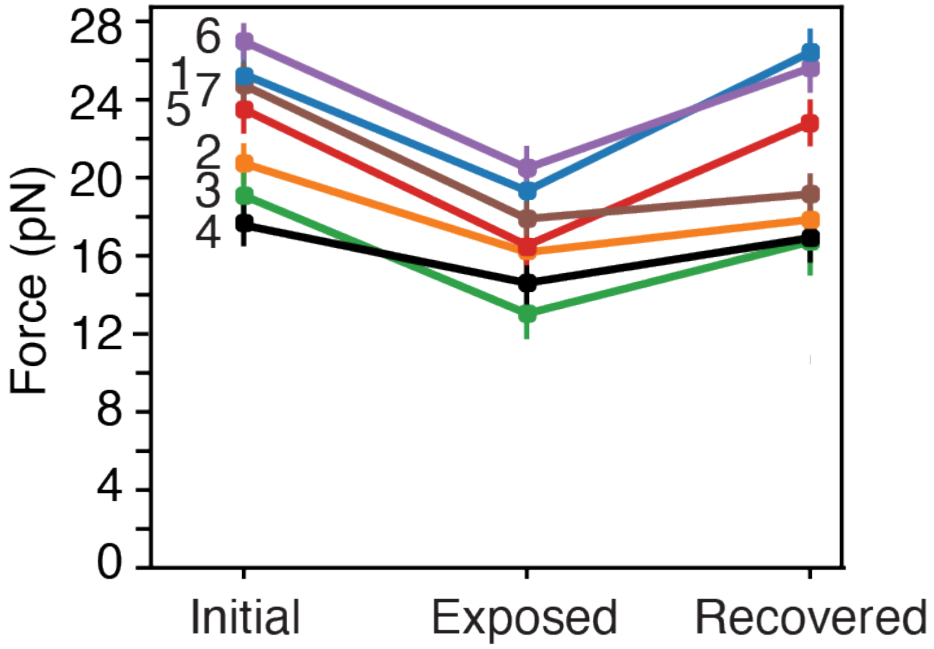

Fig. S2. Forces measured on seven cells from the bullfrog's sacculus during displacement-clamp measurements. In each instance, the bundle was driven sinusoidally through a distance of ±30 nm before, immediately following, and at least 6 s after the iontophoretic pulse. The force provided by the clamp is shown for bundles held in their resting positions (*Initial*), following the application of EDTA (*Exposure*), and at the experiment's end (*Recovered*). The data show a significant decrease ($P < 0.01$ by a single-sided paired *t*-test) in the force necessary to move the bundle after chelation, followed by a significant recovery ($P < 0.05$ by the same test) towards the initial value.

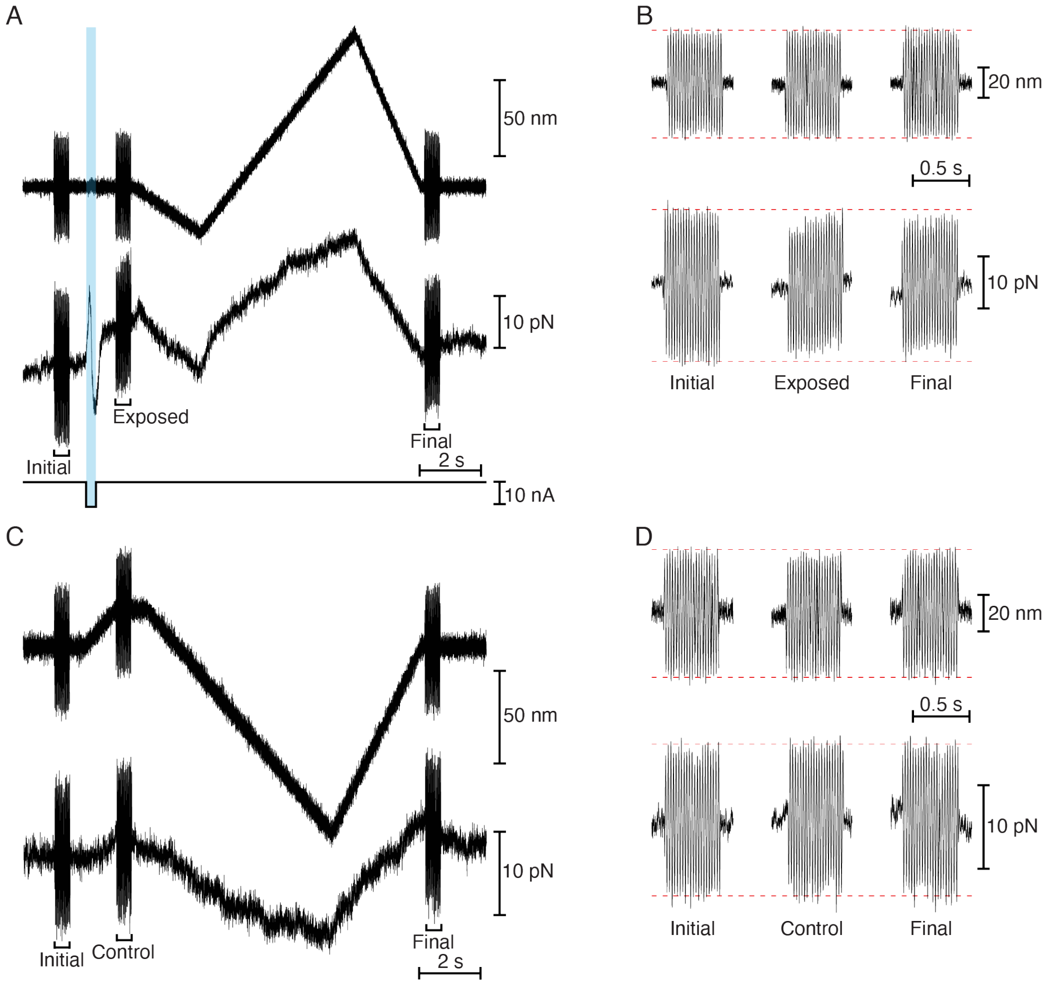

Fig. S3. Control experiments without tip-link recovery in hair bundles of the bullfrog's sacculus. (*A*) In a displacement-clamp protocol with a biphasic, predominantly positive displacement ramp (top trace) following the EDTA pulse (bottom trace), the force (middle trace) necessary to clamp the hair bundle to the desired position at the outset (*Initial*) decreased after iontophoresis (*Exposed*), but displayed no recovery after the ramp (*Final*). (*B*) Enlarged records of hair-bundle position (top traces) and force (bottom traces) confirm the decrease in hair-bundle stiffness and the failure of recovery after a positive ramp. (*C*) A hair bundle's position (top trace) and force (bottom trace) during sinusoidal stimulation (*Initial*) revealed no decrease in the stiffness in the absence of iontophoresis (*Control*) or after the ramp (*Final*). (*D*) Enlarged records of hair-bundle position (top traces) and force (bottom traces) reveal no change in hair-bundle stiffness in the absence of an iontophoretic pulse.

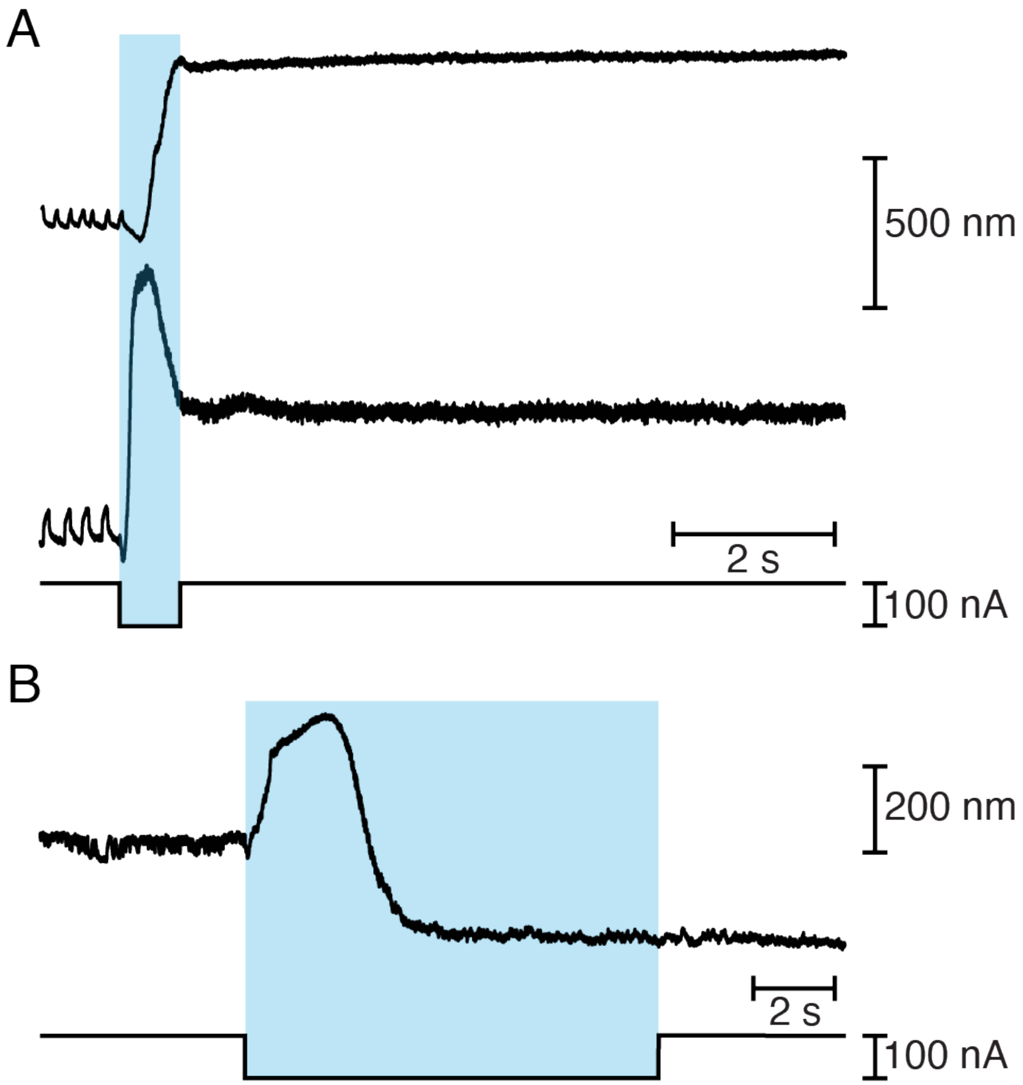

Fig. S4. Negative hair-bundle movements after Ca^{2+} chelation in the bullfrog's sacculus. (*A*) Two unrestrained, oscillating hair bundles displayed distinct responses to Ca^{2+} chelation. After a brief negative transient, one bundle (top trace) remained stationary at a large positive offset. The second hair bundle (bottom trace) initially followed a similar trajectory, but then underwent a sustained movement back in the negative direction. (*B*) In a similar experiment with a longer exposure to Ca^{2+} chelator, a bundle displayed a large negative movement after the initial positive movement and reached a plateau while iontophoresis was still in progress.

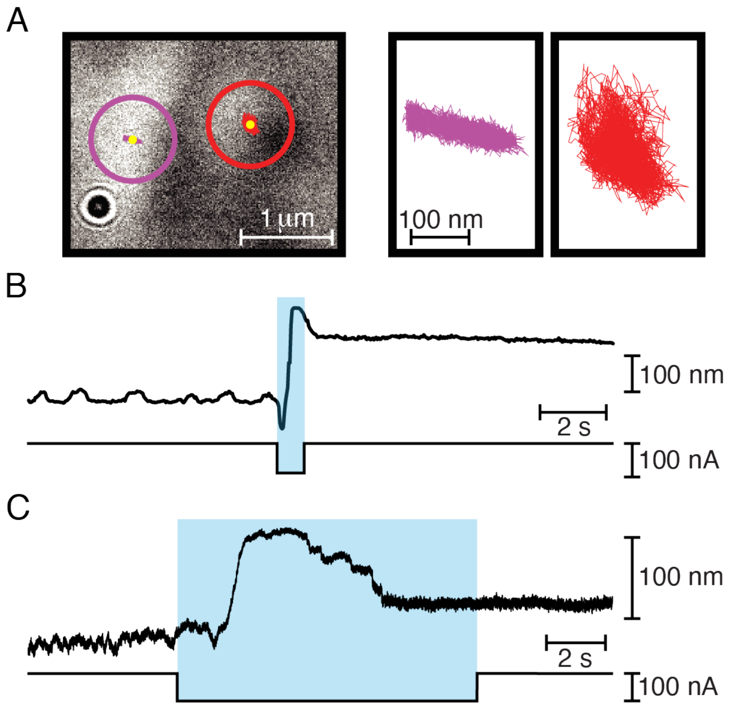

Fig. S5. Effect of dissecting kinocilia from bullfrog saccular hair bundles. (*A*) A panel from a video record (left panel) shows the top of a hair bundle whose kinocilium had been dissected free of the stereociliary cluster. The purple circle marks the area in which stereociliary motion was tracked for 10 s at 500 frames per second and the red circle the corresponding area for the kinociliary bulb. The trajectories of the respective centroids are shown under the yellow dots at the centers of the circles. Enlarged trajectories (right panels) demonstrate that the stereocilia (purple) continued to oscillate along the bundle's axis of mirror symmetry, whereas the kinocilium (red) underwent random motion. The scale bar at the right applies to both panels. (*B*) A record of 19 s of tracking at 30 frames per second (top trace) reveals the trajectories of a stereociliary cluster after kinociliary dissection. The rupture of tip links by iontophoresis of EDTA (bottom trace) elicited a conventional bipartite response followed by a negative movement. (*C*) In a similar experiment, the kinocilium was not only separated from the stereociliary cluster, but also held against the epithelial surface with a microelectrode. In this instance the negative motion occurred in several discrete steps, a phenomenon observed only after kinociliary dissection.

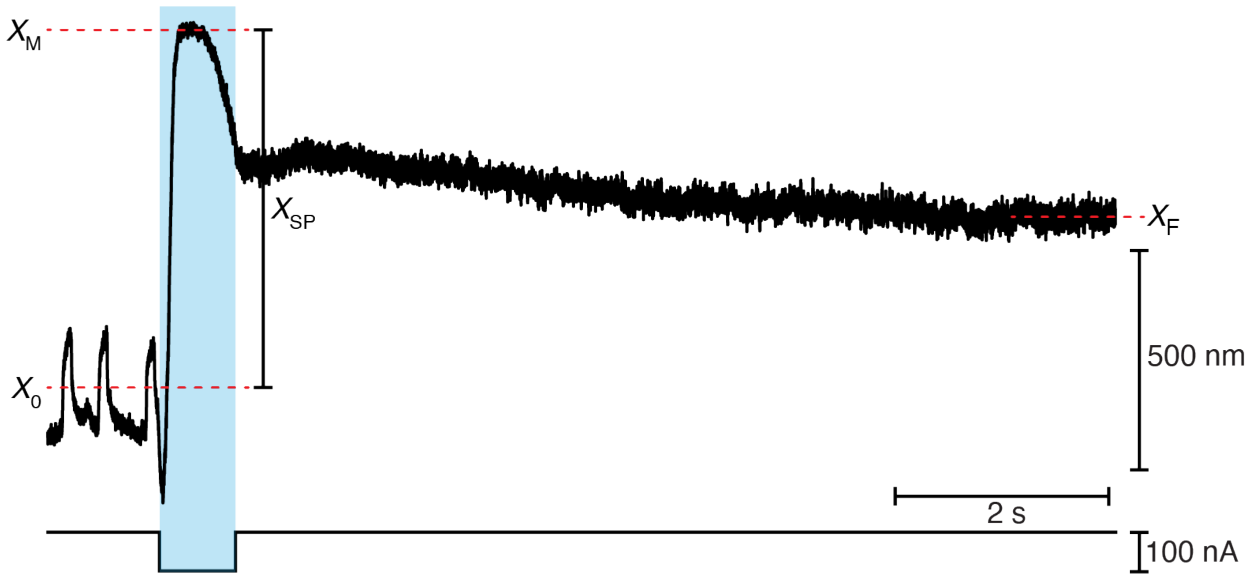

Fig. S6. Estimation of resting tip-link tension and negative movement in hair bundles from the bullfrog's sacculus. In a representative trace of an unrestrained, oscillating hair bundle, the bundle's movement upon exposure to Ca^{2+} chelator (X_{SP}) was measured from the midpoint (X_0) between the maxima and minima of the spontaneous oscillations to the maximal excursion (X_M) during the iontophoretic step. The final position (X_F) represented the average position of the hair bundle over the last second of the experiment.

SI Video Caption

Video S1. Recovery of oscillations after iontophoresis of a Ca^{2+} chelator. Viewed from above, a hair bundle from the bullfrog's sacculus displays low-frequency spontaneous oscillations. When EDTA is expelled from the pipette at the upper left, the bundle jumps in the positive direction, to the right, and ceases to move. After the metal-coated stimulus fiber at the upper right applies force in the negative direction and is then withdrawn, the bundle resumes oscillations indicative of an intact transduction process.

SI References

1. M. Tobin, A. Chaiyasitdhi, V. Michel, N. Michalski, P. Martin, Stiffness and tension gradients of the hair cell's tip-link complex in the mammalian cochlea. *eLife* **8**, e43473 (2019).

2. H. J. Kennedy, M. G. Evans, A. C. Crawford, R. Fettiplace, Fast adaptation of mechanoelectrical transducer channels in mammalian cochlear hair cells. *Nat. Neurosci.* **6**, 832–836 (2003).

3. J. B. Azimzadeh, J. D. Salvi, Physiological preparation of hair cells from the sacculus of the American bullfrog. *J. Vis. Exp.* 55380 (2017).

4. A. S. Kozlov, T. Risler, A. J. Hudspeth, Coherent motion of stereocilia assures the concerted gating of hair-cell transduction channels. *Nat. Neurosci.* **10**, 87–92 (2007).

5. J. D. Salvi, D. Ó Maoiléidigh, B. A. Fabella, M. Tobin, A. J. Hudspeth, Control of a hair bundle's mechanosensory function by its mechanical load. *Proc. Natl. Acad. Sci. U. S. A.* **112**, E1000-1009 (2015).

6. F. Jaramillo, A. J. Hudspeth, Displacement-clamp measurement of the forces exerted by gating springs in the hair bundle. *Proc. Natl. Acad. Sci. U. S. A.* **90**, 1330–1334 (1993).

7. P. Martin, A. D. Mehta, A. J. Hudspeth, Negative hair-bundle stiffness betrays a mechanism for mechanical amplification by the hair cell. *Proc. Natl. Acad. Sci. U. S. A.* **97**, 12026–12031 (2000).

8. V. Bormuth, J. Barral, J.-F. Joanny, F. Jülicher, P. Martin, Transduction channels' gating can control friction on vibrating hair-cell bundles in the ear. *Proc. Natl. Acad. Sci. U. S. A.* **111**, 7185–7190 (2014).

9. R. A. Eatock, D. P. Corey, A. J. Hudspeth, Adaptation of mechanoelectrical transduction in hair cells of the bullfrog's sacculus. *J. Neurosci.* **7**, 2821–2836 (1987).

10. J. A. Assad, G. M. G. Shepherd, D. P. Corey, Tip-link integrity and mechanical transduction in vertebrate hair cells. *Neuron* **7**, 985–994 (1991).

11. R. Marquis, A. Hudspeth, Effects of extracellular Ca^{2+} concentration on hair-bundle stiffness and gating-spring integrity in hair cells. *Proc. Natl. Acad. Sci.* **94**, 11923–11928 (1997).

12. A. J. Hudspeth, Mechanoelectrical transduction by hair cells in the acousticolateralis sensory system. *Annu. Rev. Neurosci.* **6**, 187–215 (1983).

13. R. A. Jacobs, A. J. Hudspeth, Ultrastructural correlates of mechanoelectrical transduction in hair cells of the bullfrog's internal ear. *Cold Spring Harb. Symp. Quant. Biol.* **55**, 547–61 (1990).

14. C. Spoon, W. Grant, Biomechanics of hair cell kinocilia: Experimental measurement of kinocilium shaft stiffness and base rotational stiffness with Euler-Bernoulli and Timoshenko beam analysis. *J. Exp. Biol.* **214**, 862–870 (2011).

15. R. E. Bowen, Movement of the so-called hairs in the ampullar organs of fish ears. *Proc. Natl. Acad. Sci. U. S. A.* **17**, 192–194 (1931).

16. A. Rüsch, U. Thurm, Spontaneous and electrically induced movements of ampullary kinocilia and stereovilli. *Hear. Res.* **48**, 247–263 (1990).

17. A. J. Hudspeth, R. Jacobs, Stereocilia mediate transduction in vertebrate hair cells. *Proc. Natl. Acad. Sci. U. S. A*. **76,** 1506–1509 (1979).

18. J. Y. Tinevez *et al.*, TrackMate: An open and extensible platform for single-particle tracking. *Methods* **115**, 80–90 (2017).
